# The Adoption of Viral Capsid-Derived Virus-Like Particles (VLPs) for Disease Prevention and Treatments

**DOI:** 10.3390/vaccines8030432

**Published:** 2020-08-02

**Authors:** Giorgio Bogani, Francesco Raspagliesi, Antonino Ditto, José de la Fuente

**Affiliations:** 1 Fondazione IRCCS Istituto Nazionale dei Tumori di Milano, 20133 Milan, Italy; raspagliesi@istitutotumori.mi.it (F.R.); antonino.ditto@istitutotumori.mi.it (A.D.); 2 SaBio. Instituto de Investigación en Recursos Cinegéticos, IREC (CSIC,UCLM,JCCM) Ronda de Toledo s/n, 13005 Ciudad Real, Spain; jose_delafuente@yahoo.com; 3 Department of Veterinary Pathobiology, Center for Veterinary Health Sciences, Oklahoma State University, Stillwater, OK 74078, USA

In the present paper, Mohosen et al. [1] reviewed the evidence of the interaction between virus-like particles (VLPs) and the innate immune system. VLPs are molecules resembling viruses. VLPs do not contain the genetic material of virus, and they are not infective. VLPs are an important class of nanoparticles characterized by a number of noteworthy applications into research and clinical practice. Generally, VLPs are adopted as vaccines. VLPs contain repetitive, high-density displays of viral surface proteins that present conformational viral epitopes that can elicit strong T cell and B cell immune responses [2]. Moreover, VLPs would be adopted for delivery of genes and other therapeutic agents [3]. As indicated by Mohosen et al. [1], size and surface geometry are the two main factors driving the interaction between VLPs and the humoral immune system. Additionally, the highly repetitive surface of VLPs is on the basis of the interaction with the innate immune system [4]. Another important feature in the field of VLPs, was the adoption of lipoparticles. Lipoparticles are purified and homogeneous VLPs that are engineered to contain the intact membrane proteins and are useful for delivering target therapies [5]. Furthermore, VLPs displaying the alpha-Gal carbohydrate, a molecule eliciting protective immune response against multiple pathogens [6], have shown protection against Leishmaniosis [7]. To date, there is an emerging need to develop and implement VLPs. Engineered VLPs have a role in developing new, safe vaccines and delivering (as vector) components into the cells and their membranes. Understanding the mechanisms of interaction between VLPs and the immune system would be of paramount importance. Using tumor-associated antigens, the VLPs would also be adopted for the design of anticancer vaccines (therapeutic vaccines). To date, the growing adoption of VLPs in preclinical and clinical studies aimed to bridge the gap of preventing and treating diseases, including cancer. Prophylactic vaccines against human papillomavirus (HPV) are based on VLPs. These vaccines prevent preneoplastic lesions of the female and male lower genital tract and the head and neck district [2]. Currently, some of the vaccine projects on phase I and phase II clinical trials for the control of COVID-19 are based on VLPs [8,9], thus supporting the potential for these interventions. Similarly, ongoing trials on therapeutic vaccines are based on VLPs. One of these therapeutic vaccines include the use of VLP-encapsulated TLR9 agonist, CMP-001 [7]. CMP-001 is made up of a short piece of DNA that is packaged in a VLP. The DNA contained in CMP-001 activates the immune system and recruits cells of the immune system to the tumor. INCAGN01949 is an antibody—a type of protein—which has been shown to stimulate the immune system. Injecting both CMP-001 and INCAGN01949 would reduce tumor growth [10]. Further in vitro and in vivo studies are warranted in order to clarify the mechanism of interaction between VLPs and the humoral immune system, potentially with Human Leucocyte Antigens.

(HLA) polymorphisms. Growing attempts are necessary to adopt emerging prophylactic and therapeutic vaccines for the prevention and control of major infectious and noninfectious diseases.
